# Total arthroplasty with Ivory^®^ prosthesis versus resection–suspension arthroplasty: a retrospective cohort study on 82 carpometacarpal-I osteoarthritis patients over 4 years

**DOI:** 10.1186/s40001-020-00411-8

**Published:** 2020-04-15

**Authors:** Stefan M. Froschauer, Matthias Holzbauer, Richard F. Schnelzer, Manfred Behawy, Oskar Kwasny, Matthias M. Aitzetmüller, Hans-Günther Machens, Dominik Duscher

**Affiliations:** 1 Department for Trauma Surgery and Sport Traumatology, Kepler University Hospital Linz, Medcampus III, Krankenhausstrasse 3, 4020 Linz, Austria; 2 Department for Plastic and Hand Surgery, Technical University Munich, Ismaninger Strasse 22, 81675 Munich, Germany; 3 Section for Plastic and Reconstructive Surgery, Kepler University Hospital Linz, Medcampus III, Krankenhausstrasse 3, 4020 Linz, Austria; 4 Medical Faculty, Johannes Kepler University Linz, Krankenhausstrasse 9, 4020 Linz, Austria

**Keywords:** Carpometacarpal-I osteoarthritis, Prosthesis, Implant, Ivory carpometacarpal-I prosthesis

## Abstract

**Background:**

To elucidate the performance of carpometacarpal-I joint prostheses in comparison with the current gold standard treatment, resection–suspension arthroplasty (RA), we conducted a study comparing outcomes of the Ivory prosthesis to those of a cohort of patients receiving RA.

**Methods:**

Initially, we had enrolled 34 prosthesis patients and 48 RA patients, of which 5 and 11 were lost to follow-up. We defined Eaton/Littler stage 3 osteoarthritis, no previous surgery, no concomitant arthrosis, no rheumatic arthritis, no history of trauma and a minimum follow-up period of 2 years as inclusion criteria. We assessed patient demographics, disability of the arm, shoulder, and hand score, pain via visual analogue scale, subjective strength of the thumb, range of motion (radial and palmar abduction and opposition), and patient satisfaction. All occurring complications were recorded.

**Results:**

Follow-up included a mean period of 4.5 years (2–7.4) in the prosthesis cohort and 4.1 years (2–6.8) in the RA group. Disability of the arm, shoulder, and hand scores, pain scores, palmar abduction and opposition, and subjective satisfaction showed no significant differences between the two cohorts. Postoperative loss of strength was significantly less in the prosthesis group (*p* = 0.01). Moreover, we were able to demonstrate better range of motion in terms of radial abduction in the prosthesis group (*p* = 0.001). The overall complication rate was significantly higher in the prosthesis cohort (41.4% vs. 10.8%) (*p* = 0.008). Nevertheless, the Ivory prosthesis group showed a survival rate of 93.1%.

**Conclusion:**

As the high complication rate is compensated by a better functional outcome (enhanced range of motion and strength), we believe that prosthesis implantation can be a reasonable treatment option for carpometacarpal-I osteoarthritis in a particular patient group.

*Level of Evidence IIIl*: Retrospective cohort study.

## Background

Traditional therapy approaches for carpometacarpal-I (CMC-I) osteoarthritis include conservative injection treatment, resection arthroplasty (RA), or arthrodesis. Although RA commonly represents the gold standard in the treatment of CMC-I osteoarthritis, there is no evidence that this surgical method is superior to others [1]. The major drawback of this method is proximal migration of the thumb, which can lead to compromised grip strength and disability [2].

Since de la Caffiniere launched the first CMC-I prothesis in 1974, another surgical option for the most surgically treated arthritic joint of the upper limb is available [3, 4]. The primary aim of total arthroplasty is to achieve anatomical reconstruction of the CMC-I joint, leading to better functional results in terms of stronger grip, faster and better pain relief, and better range of motion. However, the main drawback of this surgical technique is aseptic cup loosening, which represents the most common reason for implant failure. [5] Although many approaches have been used to improve the prothesis design, by modifying the biomechanics and choosing modern materials with minimal wear, literature still reports revision rates of 42%51% [6–8]. Therefore, several surgeons are still skeptical about prosthesis implantation for CMC-I osteoarthritis. Numerous theories have been raised in an attempt to explain the deficiency in several prosthesis designs, ranging from high mechanical shear forces to material bearings leading to backside wear and pseudocysts or impaired bone quality of the trapezium [9, 10].

Few studies have compared CMC-I protheses to RA [2, 11, 12]. Therefore, the aim of this retrospective study was to conduct a mid-term comparison between patients who had undergone Ivory prothesis implantation and RA.

## Methods

In this study, we included patients who underwent either RA or Ivory prosthesis implantation for primary CMC-I osteoarthritis at our institution between January 2011 and December 2015. Furthermore, an Eaton/Littler stage 3 osteoarthritis and a follow-up period of minimum 2 years were the prerequisites for inclusion in the study cohort. We excluded patients with rheumatoid arthritis, any history of trauma (e.g., Rolando fracture or Bennett’s fracture–dislocation), concomitant scapho-trapezio-trapezoid (STT) osteoarthritis, and previous surgery of the CMC-I joint. Demographic factors such as age, gender and side of CMC-I osteoarthritis were noted.

**Fig. 1:**
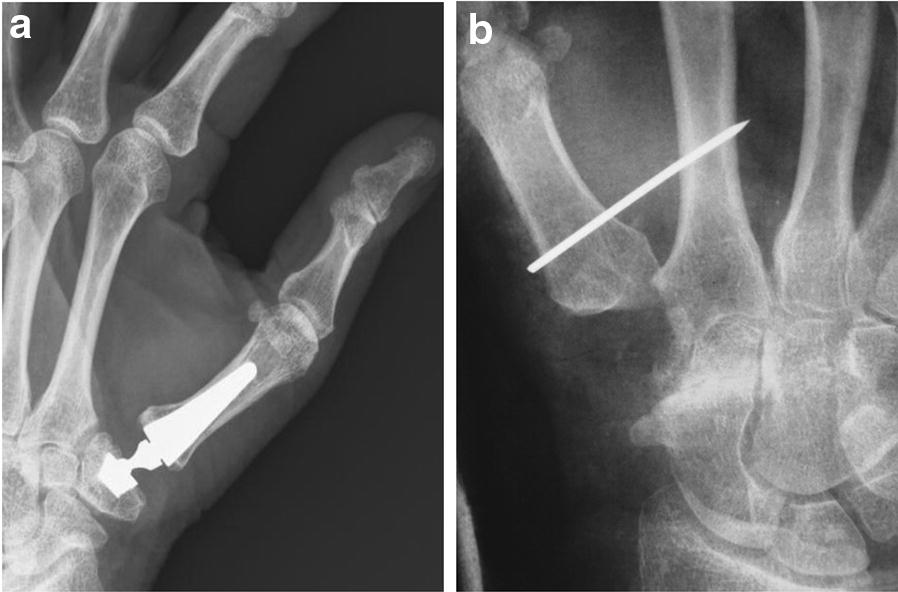
X-ray images after Ivory prosthesis implantation (**a**) and resection arthroplasty (**b**)

All surgical procedures were performed under plexus anesthesia and using a tourniquet by a senior hand surgeon assisted by a resident surgeon. For RA, we employed a technique that included restoration of the ligament connection between the first and second metacarpals after extirpation of the trapezium. We used a dorso-radial incision beginning at the base of the thumb centered over the trapeziometacarpal joint. Using blunt dissection, the radial nerve and artery and their branches were identified and protected. Then, using sharp dissection, the capsules of the trapeziometacarpal joint and the STT joint are identified and incised longitudinally. While paying attention to the radial artery, we extracted the trapezium. Furthermore, a distally based extensor carpi radialis longus (ECRL) tendon strip (4 cm) was used for suspension and ligament reconstruction by passing it through the base of the first metacarpal and fixing it under appropriate tension via a Micro Mitek bone anchor (Johnson and Johnson, USA). To allow for tendinous healing and maintenance of reduction, a temporal arthrodesis was placed with a Kirschner wire (K-wire) through metacarpal (MC) I bone and MC II bone (Fig. 1a). Post-interventional care included 6 weeks of immobilization (splinting). The K-wire was removed at that time, and extensive hand therapy was performed for another 6 weeks.

For Ivory prosthesis (Memometal, Stryker, Michigan, USA) implantation, we made a dorsal-ulnar approach. After incising the skin, spreading dissection was carried out, and the CMC-I joint was entered via a 4-cm-long incision on the dorsal side of the thumb. Care was taken to identify and spare the branches of the radial sensory nerve and small vessels on the dorsal radial aspect. We opened the capsule longitudinally. Next, the MC I bone was mobilized, and an osteotomy was performed 0.5 cm distal to the joint surface to resect the MC I base. The prosthesis shaft was placed under X-ray control after adequate reaming of the metacarpal canal. Subsequently, the trapezium was mobilized, and a plane surface was generated by performing an osteotomy of the saddle-shaped trapezial joint. In the center of the trapezium, a recess was created for the implant, and the socket was inserted via a press-fit mechanism under X-ray control. Ultimately, the polyethylene-inlay was placed; the prosthesis head was inserted; and the components were evaluated for alignment, tissue tension, and joint stability **(**Fig. 1b**)**. Post-interventional care included 3 weeks of immobilization (splinting), followed by intensive hand therapy for 6 weeks.

Follow-up included at least 8 appointments, wherein radiographs in two planes (anteroposterior and lateral) were evaluated each time. At least 2 years postoperatively, we conducted a detailed clinical assessment. We evaluated the disability of the arm, shoulder, and hand (DASH) score and the visual analogue scale (VAS) for assessment of pain in both groups [13]. We assessed how the surgery had affected the subjective strength of the thumb 1 year after surgery. The strength scale ranged from -2 to +2, with –2 suggesting that the thumb is unable to grasp anything, 0 representing no noticeable change after surgery, and +2 indicating significantly enhanced force. Additionally, the range of motion (radial and palmar abduction) was measured. We also evaluated patient satisfaction and asked whether they would undergo this procedure again. Moreover, in the RA patients, proximalization of the thumb was analyzed by comparing the intraoperative radiographs with the ones taken at our final follow-up appointment, where we also evaluated all parameters presented above. All occurring complications were recorded.

Statistical analysis was performed using SPSS (version 24.0). Descriptive statistics are presented as median and interquartile range, because all our data are non-normal. For comparison of groups, Mann–Whitney U test and Fisher’s exact test were performed. A value of *p* < 0.05 was considered significant.

## Results

We had included 48 patients in the RA group and 34 patients in the prosthesis group; however, 11 and 5 patients of the RA and prosthesis groups, respectively, were lost to follow-up and had to be excluded. Patient demographics are shown in Table 1. Average follow-up period was 4.1 years (2–6.8 years) in the RA group and 4.5 years (2–7.4 years) in the prosthesis group.

**Table 1:** Patient demographics

Demographics	Prosthesis implantation	Resection arthroplasty
Total	29	37
Male/female	5/24	7/30
Mean age (range)	54.4 years (45–71 years)	60.9 years (47–74 years)
Right/left	15/14	17/20

Postoperative DASH scores showed no significant differences between procedures. While RA led to a median postoperative DASH score of 30.0 (37), prosthesis implantation resulted in a DASH score of 17.5 (17) (*p* = 0.22). The Ivory prosthesis group showed slightly higher VAS values than the RA group, with median scores of 3 [5] and 1 [3], respectively (*p* = 0.07). When evaluating the subjective loss of strength, we found significantly better function in the prosthesis group. While median loss of strength was-1 (0) in the RA group, we detected diminished postoperative strength of-1 (1) in prosthesis patients (*p* = 0.01). In addition to improved strength, we could also demonstrate better range of motion (ROM) in the prosthesis group. For radial abduction, median ROM was 53.8° (10°)in the RA group, whereas the prosthesis group showed a significantly higher median ROM (61.4° (10°)) (*p* = 0.001). Median palmar abduction, with 55.4° (10°) in the RA group, showed a trend to be better in prosthesis patients (57.9° (10°)) (*p* = 0.07). Concerning opposition, we could not detect any difference between the two cohorts. Median opposition of RA patients was 0 cm (1 cm), whereas prosthesis implantation resulted in median opposition of 0 cm (1 cm) (*p* = 0.76). Outcomes are summarized in Table 2.

**Table 2:** .

Evaluation	DASH Score*	VAS Score*	Subjective strength*	Radial abduction*	Palmar abduction*	Opposition*	Patients satisfaction	Complication rate
Prosthesis implantation (*n* = 29)	17.5 (17)	1 (3)	− 1 (1)	61.4° (10°)	57.9° (10°)	0 cm (1)	96.6%	41.4%
Resection arthroplasty (*n* = 37)	30.0 (37)	3 (5)	− 1 (0)	53.8° (10°)	55.4° (10°)	0 cm (1)	91.9%	10.8%
*p* value**	0.22	0.07	*0.01*	*0.001*	0.07	0.76	0.63	*0.008*

*Values are presented as median (interquartile range)

**Mann–Whitney U test was used for DASH scores, VAS scores and mobility. Fisher’s exact test was used for patient satisfaction and complication rate

Statistically significant *p*-values are printed in italics

Regarding complications, in the RA group, four major complications occurred among four patients, including two complex regional pain syndromes (CRPS), one infection, and one massive proximalization of the thumb. Regarding the proximalization of the thumb, we could detect a median proximalization of 3 mm (0–12 cm), whereas we defined a value of more than 10 mm as complication. In the prosthesis group, 12 complications occurred among nine patients. Five Patients developed de Quervain syndrome, and in one case, the nervus radialis was injured. The prosthesis luxated in four patients, leading to revision surgery in all cases. In three cases, a change in neck length led to a stable situation, whereas one of these patients needed to be surgically converted into an RA after he presented a surgical site infection after neck length change. In one case, a prosthesis explantation and surgical conversion into an RA needed to be performed after a prothesis luxation. Therefore, the overall complication rate was significantly higher in the prosthesis group (41.4%) than in the RA group (10.8%) (*p* = 0.008). The Ivory prosthesis shows a survival rate of 93.1% in our study cohort.

The patient satisfaction analysis revealed that 96.6% of the prosthesis group were satisfied with the treatment, whereas 91.9% of the RA group would undergo treatment again. Thus, no significant difference in subjective satisfaction was observed between both groups (*p* = 0.63).

## Discussion

The present retrospective cohort study comparing Ivory prosthesis implantation and RA shows significantly higher performance scores in the prosthesis cohort with respect to radial abduction and subjective loss of strength. Moreover, we could detect a favorable trend concerning better VAS and radial abduction in the prosthesis group. These advantages of prosthesis may result from a more anatomical reconstruction of the thumb MC joint. Other comparative studies corroborate our findings and even present better results in every performance parameter for prosthesis implantation [2, 11, 12]. Cebrian-Gomez et al. also included Ivory prosthesis patients in their comparative survey with a comparable clinical follow-up period. While the functional outcomes reported by them are very similar to our results, the complication rate of 41.4% in our study is considerably higher. Regarding complications, the overall complication rate in our study seems to be more comparable to those in the early days of CMC-I total arthroplasty, where aseptic cup loosening was the major problem [9]. However, the main postoperative difficulty of our prosthesis cohort was less about osteointegration and more about tendinous structures. Because we identified de Quervain syndrome as the most frequent complication, we hypothesize that the prothesis implantation modifies the physiological direction of movement of tendons inserting into the thumb. Especially the abductor pollicis longus and extensor pollicis brevis tendons seem to become vulnerable in the first dorsal compartment after prosthesis implantation. De Quervain syndrome is also the most frequent complication (10%) in the study by Závodský, which reports short-term results of Ivory prosthesis [14].

However, the overall survival rate (93.1%) observed in our study is very similar to those reported by other studies concerning Ivory prosthesis in a mid-term follow-up period—96% by Cebrian-Gomez et al., 95% by Goubau and Goorens et al., and 85% by Spaans, van Minnen et al. [11, 15, 16]. The subjective patient satisfaction (96.6%) correlates with the high survival rate (93.1%) observed in the present study.

The present study includes several limitations. Our assessment contained a number of subjective parameters such as subjective grip strength. Moreover, we were only able to present follow-up data owing to a lack of preoperative data. Because the present study reports mid-term sustainability of both methods, we are already collecting data for a long-term evaluation.

In conclusion, we recommend Ivory prosthesis for young and active patients considering its favorable functional outcomes. Carpometacarpal-I total arthroplasty can become a reasonable therapeutic option for thumb CMC arthrosis surgery, especially if we can overcome the hurdle of high complication rates. Therefore, randomized studies with mid- or long-term follow-up are needed to verify sustainability of these prostheses.

## Data Availability

The datasets used and analyzed during the present study are available from the corresponding author on reasonable request.
